# Supplementary material for: The Systems Biology Research Tool: evolvable open-source software
Source: BMC Syst Biol. 2008 Jun 29;2:55. doi: 10.1186/1752-0509-2-55 (PMC2446383; doi:10.1186/1752-0509-2-55)
Supplement: Additional file 1 — SBRT Archive. An archive of the current version of the Systems Biology Research Tool. [file 1752-0509-2-55-S1.zip › sbrt-1.4.0/doc/developers_guide/api/sbrt/shell/proc/fba/PalssonSbmlReaderManager.html]

PalssonSbmlReaderManager


---


|  |  |  |
| --- | --- | --- |
| **Overview** \| **Package** \| **Class** \| **Use** \| **Tree** \| **Deprecated** \| **Index** \| **Help** | |  |
| **PREV CLASS**   **NEXT CLASS** | **FRAMES**    **NO FRAMES**     **All Classes** |  |
| SUMMARY: NESTED \| FIELD \| CONSTR \| METHOD | DETAIL: FIELD \| CONSTR \| METHOD |  |


---


## sbrt.shell.proc.fba Class PalssonSbmlReaderManager

```
java.lang.Object
  sbrt.shell.mng.AbstractProcessManager
      sbrt.shell.proc.fba.PalssonSbmlReaderManager
```

**All Implemented Interfaces:**: ProcessManager<java.lang.String,java.lang.String>, ShellProcessManager<java.lang.String,java.lang.String>

---

``` public final class PalssonSbmlReaderManager extends AbstractProcessManager implements ShellProcessManager<java.lang.String,java.lang.String> ```

This class is used to manage the reading of SBML files obtained from Palsson's
website.

**Author:**
:   This class was written and documented by Jeremiah Wright while in the
    Wagner lab.

---

| **Field Summary** | |
| --- | --- |
| `static java.lang.String` | `FLUX_BOUNDS_FILE_NAME`             Used to denote the name of the file to which the flux bounds should be written. |
| `static java.lang.String` | `FLUX_VECTOR_FILE_NAME`             Used to denote the name of the file to which the flux vector should be written. |
| `static java.lang.String` | `GENE_FILE_NAME`             Used to denote the name of the file to which gene-reaction relationships should be written. |
| `static UnmodifiableSet<java.lang.String>` | `optionalKeywords`             The set of keywords understood, but not required, by this process manager. |
| `static java.lang.String` | `PROTEIN_FILE_NAME`             Used to denote the name of the file to which protein-reaction relationships should be written. |
| `static UnmodifiableSet<java.lang.String>` | `requiredKeywords`             The set of keywords required by this process manager. |
| `static java.lang.String` | `RXN_FILE_NAME`             Used to denote the name of the file to which chemical reactions should be written. |
| `static java.lang.String` | `SBML_INPUT_FILE`             The keyword used to denote the name of the SBML input file. |

**Fields inherited from class sbrt.shell.mng.AbstractProcessManager**

`ALGORITHM, COMPLETED, DATA_HEADERS, DEV_NULL, ELAPSED_TIME, FILE_FORMAT, INPUT_FILE, INPUT_FILE_NAME_FILE, ITERATIONS, OUTPUT_FILE_NAME, OUTPUT_FILE_NAME_FILE, PERCENT, PERCENT_COMPLETED, SEED, ZERO_CUTOFF`


**Constructor Summary**

`PalssonSbmlReaderManager()`


| **Method Summary** | |
| --- | --- |
| `void` | `execute(java.io.PrintWriter out)`             Executes the process this process manager controls and writes informative messages to the provided print writer. |
| `UnmodifiableSet<java.lang.String>` | `getOptionalKeys()`             Returns the set of keywords understood, but not required, by this process manager. |
| `UnmodifiableSet<java.lang.String>` | `getRequiredKeys()`             Returns the set of keywords required by this process manager. |
| `void` | `setInput(ManagerInput<java.lang.String,java.lang.String> input, java.io.PrintWriter out)`             Uses the provided input to prepare this process manager for execution. |

**Methods inherited from class sbrt.shell.mng.AbstractProcessManager**

`check, closeFile, closeRm, execute, getFileFormat, getFileFormat, getFileNameSource, getIterations, getSeed, getTextFileFormat, getZeroCutoff, getZeroCutoff, parseInt, parseLong, printProgress, printProgress, printProgress, runProcess, setInput`

**Methods inherited from class java.lang.Object**

`clone, equals, finalize, getClass, hashCode, notify, notifyAll, toString, wait, wait, wait`

**Methods inherited from interface sbrt.shell.mng.ProcessManager**

`check, execute, setInput`

**Field Detail**

### GENE\_FILE\_NAME

```
public static final java.lang.String GENE_FILE_NAME
```

:   Used to denote the name of the file to which gene-reaction relationships
    should be written.

    **See Also:**: Constant Field Values

---


### PROTEIN\_FILE\_NAME

```
public static final java.lang.String PROTEIN_FILE_NAME
```

:   Used to denote the name of the file to which protein-reaction relationships
    should be written.

    **See Also:**: Constant Field Values

---


### RXN\_FILE\_NAME

```
public static final java.lang.String RXN_FILE_NAME
```

:   Used to denote the name of the file to which chemical reactions should be written.

    **See Also:**: Constant Field Values

---


### FLUX\_VECTOR\_FILE\_NAME

```
public static final java.lang.String FLUX_VECTOR_FILE_NAME
```

:   Used to denote the name of the file to which the flux vector should be written.

    **See Also:**: Constant Field Values

---


### FLUX\_BOUNDS\_FILE\_NAME

```
public static final java.lang.String FLUX_BOUNDS_FILE_NAME
```

:   Used to denote the name of the file to which the flux bounds should be written.

    **See Also:**: Constant Field Values

---


### SBML\_INPUT\_FILE

```
public static final java.lang.String SBML_INPUT_FILE
```

:   The keyword used to denote the name of the SBML input file.

    **See Also:**: Constant Field Values

---


### requiredKeywords

```
public static final UnmodifiableSet<java.lang.String> requiredKeywords
```

:   The set of keywords required by this process manager.

---


### optionalKeywords

```
public static final UnmodifiableSet<java.lang.String> optionalKeywords
```

:   The set of keywords understood, but not required, by this process
    manager.


**Constructor Detail**

### PalssonSbmlReaderManager

```
public PalssonSbmlReaderManager()
```


**Method Detail**

### getRequiredKeys

```
public UnmodifiableSet<java.lang.String> getRequiredKeys()
```

:   Returns the set of keywords required by this process manager.

    :   **Specified by:**: `getRequiredKeys` in interface `ProcessManager<java.lang.String,java.lang.String>`
    :   **Returns:**: the set of keywords required by this process manager.

---


### getOptionalKeys

```
public UnmodifiableSet<java.lang.String> getOptionalKeys()
```

:   Returns the set of keywords understood, but not required, by this process
    manager.

    :   **Specified by:**: `getOptionalKeys` in interface `ProcessManager<java.lang.String,java.lang.String>`
    :   **Returns:**: the set of keywords understood, but not required, by this process manager.

---


### setInput

```
public void setInput(ManagerInput<java.lang.String,java.lang.String> input,
                     java.io.PrintWriter out)
```

:   Uses the provided input to prepare this process manager for execution. As
    the input is processed, informative messages are printed to the provided
    print writer.

    :   **Specified by:**: `setInput` in interface `ProcessManager<java.lang.String,java.lang.String>`
    :   **Parameters:**: `input` - all of the information required to prepare this process manager for execution.: `out` - the print writer to which all messages will be printed.

---


### execute

```
public void execute(java.io.PrintWriter out)
```

:   Executes the process this process manager controls and writes informative messages
    to the provided print writer.

    :   **Specified by:**: `execute` in interface `ProcessManager<java.lang.String,java.lang.String>`
    :   **Parameters:**: `out` - the print writer where informative messagse will be written. **Throws:**: `java.lang.IllegalStateException` - if no input was provided to this process manager.


---


|  |  |  |
| --- | --- | --- |
| **Overview** \| **Package** \| **Class** \| **Use** \| **Tree** \| **Deprecated** \| **Index** \| **Help** | |  |
| **PREV CLASS**   **NEXT CLASS** | **FRAMES**    **NO FRAMES**     **All Classes** |  |
| SUMMARY: NESTED \| FIELD \| CONSTR \| METHOD | DETAIL: FIELD \| CONSTR \| METHOD |  |


---
